# Remote Cognitive Assessment for Preoperative Evaluation in Older Adults: Feasibility and Associations with Subjective Cognitive Concerns

**DOI:** 10.1002/alz.094359

**Published:** 2025-01-09

**Authors:** Amy B. Wise, Sreya Dhanam, Sara Zhou, Anika Sinha, Kaylee Hernandez, Niti Pawar, Adam L. Boxer, Odmara L Barreto Chang, Adam M. Staffaroni

**Affiliations:** ^1^ Memory and Aging Center, UCSF Weill Institute for Neurosciences, University of California, San Francisco, San Francisco, CA USA; ^2^ University of California, San Francisco, San Francisco, CA USA; ^3^ Memory and Aging Center, Weill Institute for Neurosciences, University of California, San Francisco, San Francisco, CA USA

## Abstract

**Background:**

Postoperative complications of major surgical interventions include delirium. Delirium is a risk factor for dementia, and in some cases, may signal underlying neuropathological processes. Cognitive tests that accurately predict post‐operative outcomes could identify patients with cognitive vulnerabilities who may benefit from preoperative counseling and postoperative interventions. Unfortunately, in‐person presurgical cognitive evaluations are often not available, but digital technologies may improve access. In this pilot study, we evaluate the feasibility of utilizing a smartphone app to conduct preoperative remote cognitive assessment in patients undergoing spine surgery. We also evaluate the relationship of app‐based assessments with subjective cognitive concerns.

**Method:**

We enrolled 30 older adults ( = 65 years old) who were scheduled for elective spinal surgery anticipated to last at least three hours. On the ALLFTD Mobile App, participants completed a battery of cognitive tests, a measure of subjective cognitive concerns (12‐item self‐reported everyday cognition scale, Ecog‐12), and a user experience survey. A subset (N = 12) had a study partner complete the 41‐item Ecog, reporting on observed changes in cognitive function over a 10‐year period prior to the surgery. Linear regressions adjusted for age evaluated the associations between subjective cognitive concerns and the objective app‐based outcomes (Flanker task and a composite of several measures).

**Result:**

Average age of participants was 71.2 years (SD = 4.4); 46.1% were women and 75% identified as White. Most (96%) participants indicated that the time required to complete the app assessments was acceptable, 77% indicated that the app instructions were clear, and all participants indicated that the text on the app was large enough to read comfortably. Participants' subjective cognitive concerns (ECog‐12) were significantly associated with a composite of app tasks (ß = ‐0.48, p = 0.01). The executive functioning domain of the study partner Ecog was significantly associated with patient performance on an app‐based executive functioning task (ß = 0.75, p = 0.007).

**Conclusion:**

These findings support the feasibility of remote preoperative assessments in older adults using smartphones. The study also provides preliminary support that these measures can objectively quantify self‐ and partner‐reported cognitive concerns. Future work will explore associations with postoperative outcomes.

